# On the Adoption of Radiomics and Formal Methods for COVID-19 Coronavirus Diagnosis

**DOI:** 10.3390/diagnostics11020293

**Published:** 2021-02-12

**Authors:** Antonella Santone, Maria Paola Belfiore, Francesco Mercaldo, Giulia Varriano, Luca Brunese

**Affiliations:** 1 Department of Medicine and Health Sciences “Vincenzo Tiberio”, University of Molise, 86100 Campobasso, Italy; luca.brunese@unimol.it; 2 Department of Precision Medicine, University of Campania “Luigi Vanvitelli”, 80138 Napoli, Italy; mariapaola.belfiore@unicampania.it

**Keywords:** radiology, radiomics, formal methods, artificial intelligence, Coronavirus, COVID-19, diagnosis, HRCT, CT

## Abstract

Considering the current pandemic, caused by the spreading of the novel Coronavirus disease, there is the urgent need for methods to quickly and automatically diagnose infection. To assist pathologists and radiologists in the detection of the novel coronavirus, in this paper we propose a two-tiered method, based on formal methods (to the best of authors knowledge never previously introduced in this context), aimed to (i) detect whether the patient lungs are healthy or present a generic pulmonary infection; (ii) in the case of the previous tier, a generic pulmonary disease is detected to identify whether the patient under analysis is affected by the novel Coronavirus disease. The proposed approach relies on the extraction of radiomic features from medical images and on the generation of a formal model that can be automatically checked using the model checking technique. We perform an experimental analysis using a set of computed tomography medical images obtained by the authors, achieving an accuracy of higher than 81% in disease detection.

## 1. Introduction

In radiological examination, the information is obtained using different techniques (such as X-rays) and thanks to analyses performed by specialists. This analysis can only be qualitative because it is performed by an experienced specialists. These subjective elements can be affected by different allusions or perceptions on the basis of the morphology of the images and on personal life experience. For these reasons, there is the need for apply methods to medical imaging that are aimed at assisting specialists with an objective analysis. Radiomics is a research field that can help to reach this goal, extracting information and data from medical images.

The proposed method is based on radiomic feature extraction: a piece of software takes the radiological exams and extracts numerical values that describe, in a quantitative way, the image [1]. It is well known that, if a feature is measurable, it will be easily managed: in fact, in the medical field, radiomic features can lead to a quick, low cost and accurate diagnosis. In this case, we use these extracted values to create a patient profile and verify if their values belong to a certain medical status. Furthermore, among thousands of features of radiomic tools and, in the absence of a radiomic feature standards, we performed an analysis to choose the best set of features to classify a medical status category.

At the end of 2019, the Coronavirus pandemic started. The virus is referred to as SARS-Cov-2 (Severe Acute Respiratory Syndrome-Coronavirus-2), which causes a disease known as COVID-19. This virus causes abnormal pneumonia, so radiological imaging plays a key role in the diagnosis, management and follow-up of the disease. The recognition of specific characteristics of the virus by pathologists and radiologists is fundamental to the detection of the disease, to the assessment of the severity of the disease, and to the correct interpretation of the follow-up until resolution. Nowadays, it is possible to carry out tests for the diagnosis of infection by SARS-Cov-2 and, at the moment, specific treatments and vaccines are being researched, tested and released. In addition to preventative testing, Computed Tomography (CT) showed features consistent with doctors’ diagnoses, but due to other similar viruses and pneumonia, the exclusive use of the CT scan could be useless for the classification of COVID-19 disease. Radiologists have the possibility to make the virus visible by choosing the appropriate radiological imaging. For instance, the standard chest Radiography exam (RX) has low sensitivity in identifying early lung changes. High Resolution Computational Tomography (HRCT), on the other side, represents the method widely chosen in the study of the virus [2], given its high sensitivity and resolution. As well as at the initial stages, the HRCT examination is also useful for monitoring radiological pictures over time, especially to elucidate the severity of the disease.

COVID-19 pneumonia presents various and nonspecific findings and patterns, which can be commonly found in other pneumonitis. The HRCT examination findings showed the “frosted glass” areas (ground glass) bilateral, which is associated with peripheral or subpleuric consolidation areas and with greater involvement of the back regions and lower lobes. Recognizing COVID-19 pneumonia is very difficult and presents obstacles in the diagnostic process, due in part to the possible inconsistency of different exams and the lack of clinical expertise needed to interpret diagnostic results in addition to the huge volume of cases to treat in the shortest time possible.

Currently, the measures adopted for testing COVID-19 infection consist in performing molecular and antigen tests, also called transcriptase-polymerase chain reaction (RT-PCR). In particular, as stated by [3], RT-PCR tests have adequate sensitivity to the diagnosis of early infections of COVID-19. However, “It is well known that results from real-time RT-PCR using primers in different genes can be affected by the variation of viral RNA sequences. Genetic diversity and rapid evolution of this novel coronavirus have been observed in different studies [4,5]. False-negative results may occur by mutations in the primer and probe target regions in the SARS-CoV-2 genome”. This is claimed by Tahamtan and Ardebili in [3]. In addition, the authors in [6] reported that many “suspected” cases with typical clinical characteristics of COVID-19 and identical specific CT images were not diagnosed. Thus, a negative result does not exclude the possibility of COVID-19 infection and should not be used as the only criterion for treatment or patient-management decisions. This can be used to support the idea of combining RT-PCR tests with other clinical features, such as CT exams, to reduce inaccurate results and improve the approach. There are a number of works that compare RT-PCR and CT exams. As asserted by [7], the use of CT to screen for COVID-19 patients is fundamental, especially when the results of RT-PCR tests are negative or doubtful in symptomatic patients. In the study of Reginelli et al. [8], the authors argued that there are pathgnomonic CT signs that are useful in monitoring the evolution of the disease and that also determine the degree of severity based on the involvement of the number of affected lung lobes. The patient’s clinic, combined with the imaging, can help in confirming the diagnosis.

The diagnosis of COVID-19 pneumonia comprise a combination of epidemiological, clinical and radiological data. Although the pattern is nonspecific, images contain typical areas and sickness markers that can be recognized by the radiologist as a general pneumonia. The role of the radiologist in this process is fundamental to the initial identification of the disease, to the assessment of severity, and in the correct interpretation of the temporal changes in the radiological framework, up to the resolution of the disease. HRCT is advantageous as it also allows for the identification of cases of incomplete resolution and possible re-infection.

For these reasons, there is currently the need for software tools aimed at assisting in the detection of pathologies from radiological image analysis. Artificial Intelligence (AI) techniques are currently being used for this purpose. This work, instead, introduces the use of formal verification techniques in this context. Formal Methods [9] are mathematical algorithms normally used for the software and hardware verification of critical and complex systems. These techniques, which allow for the precise and rigorous analysis of a system, can offset some of the current limits of AI [10]. For instance, formal methods do not require huge data sets to ensure the robustness of the model. In fact, the model is more generalizable and there is the possibility of explaining the mental process that led to the  obtained result.

This paper aims at the definition and implementation of an innovative method exploiting Formal Methods (never applied in this context, to the best of authors’ knowledge) with which, through the radiological images of the patients, it will be possible to understand the most important characteristics of COVID-19. This analysis will be performed to obtain a quick, accurate and automatic diagnosis of healthy, COVID-19 and lung disease patients through a non-invasive, fast and low-cost approach, ensuring great benefits for the medical field.

In particular, this case study takes into account three types of medical status categories: healthy, COVID-19 and lung disease patients. As already mentioned, CT coronavirus findings are nonspecific and can be confused with other pneumonitis findings and vice-versa. For this reason, the dataset was also filled with lung disease patients: they are patients with different types of pneumonitis. Once the model of the patient is drafted and the medical status property verified, the result of the method is a diagnosis: the patient can be healthy or affected by COVID-19 or by a lung disease. The accuracy of the method is improved by the building of a two-tier methodology: in each tier, the classification problem is reduced from 3 to 2 classes. This allows one to deeply concentrate on the characteristics of the diseases and to reduce the probability of errors. The reached results, indeed, confirm the idea of the authors—in each tier, the accuracy is greater or equal than 81%.

The proposed method could never replace the role of radiologists: the final result, namely the diagnosis, will be a “second virtual opinion” for the radiologist who is facing the ongoing epidemiological emergency. Additionally, with formal methods the specialists can easily enrich the models of their experience and knowledge. It can be easy to understand a mathematical model and identify patterns of a certain state of healthy. This can be an advantage compared to AI “black boxes”, that appear more difficult to explain.

## 2. Materials and Methods

In the following section, we provide background notions about formal methods, widespread adopted by the proposed approach, and we describe our proposal in detail.

### 2.1. Background about Formal Methods

People are sometimes unaware of the critical digital systems they use in everyday life. There are many critical systems, such as those that are business-critical of the Stock Exchange that must manage a million transactions per second; there are others that are life-critical, such as the Avionic Control System of a nation. In these critical contexts, bugs could be turned into economic or life losses. For these reasons, the correctness of a digital system is crucial and the security and safety tasks can be entrusted to formal methods; namely, mathematician techniques allowing one to have a formal proof of the correctness of concurrent systems. This is a systematic, mathematical and rigorous approach to verify the correct behaviour of the system.

To project and verify systems using formal methods, there exist different languages and techniques. Of all of these, the language used in this paper is the Calculus of Communicating System (CCS) [11], while the technique is Model Checking [12], which will be discussed in the next subsection. The CCS was proposed by Milner et al. [11] to produce formal models, starting with the description of the system’s behaviour. To graphically describe the system behaviour, the Labelled Transition System (LTS) is used: it is a structure using states and transitions to move from state *p* to *q* through action α. The LTS starts from a particular state called *initial* state. The CCS uses different operators and an operational semantic to automatically generate the LTS model of the system. An operational semantic manages the possible steps to perform through axioms and transition rules.

One environment of this calculus is the CWB—New Century [13], a tool that allows to model and check concurrent system behaviours.

#### Model Checking

Model Checking [12] is a profitable technique because it allows for the automatic checking of the model for a greater usage. Its goal is to verify if the model satisfies a property (or formula) ϕ. The algorithm, developed by the authors, runs with the input of the model and the property: if the output is true, the property is satisfied by the model, otherwise the output will be false. In this latter case, the Model Checker returns a counterexample that can be used to explain the process that led to a false result.

In this paper, the Model Checker will automatically verify if the patient is healthy or not according to whether the property is satisfied or not. Generally, a system implementation should respect and satisfy the system requirements, so the corresponding property will be:

implementation ⊧ systemRequirements

The implementation is a property, namely a particular aspect of the system, written through a mathematical logic that is temporal and recursive. The logic chosen for this work is the *mu-calculus* [14] (or μ-calculus), the syntax of which is below reported. It is supposed that *Z* ranges over a set of variables, and *K* and *R* range over sets of *A* actions.

$$\begin{array}{ccc} \phi & \;::=\;\; & \mathrm{tt}\;|\;\mathrm{ff}\;|\;Z\;|\;\phi \vee \phi \;|\;\phi \wedge \phi \;|\;[K]\phi \;|\;<K>\phi \;|\;\nu Z.\phi \;|\;\mu Z.\phi \end{array}$$

*Mu-calculus* is widely used to describe the LTS properties and to verify them in a temporal and recursive way. The fixed-point operators used are μZ.ϕ and νZ.ϕ, where μZ(νZ) binds free occurrences of *Z* in ϕ. An occurrence of *Z* is free if it is not within the scope of a binder μZ(νZ). From these, μZ.ϕ is the least fix-point of the recursive equation Z=ϕ, while νZ.ϕ is the greatest one. A formula is closed if it contains no free variables.

A LTS *M* satisfies a formula ϕ, denoted M⊧ϕ, if and only if q⊧ϕ, where *q* is the initial state of *M*.

The satisfaction of the formula ϕ by a state *s* of a LTS *M* is so defined:

- Each state satisfies tt and no state satisfies ff.
- A state satisfies ϕ∧ψ ( or ϕ∨ψ) if it satisfies ϕ and (or) ψ.
- [K]ϕ and <K>ϕ are modal operators, so:
  — [K]ϕ is satisfied by a state which, for each performance of an action in *K*, evolves in a state that meets ϕ.
  — <K>ϕ is satisfied if exists a state which can evolve to another state that meets ϕ by performing an action in *K*.

The Model Checking problem for this logic is “given a formula ϕ, and a LTS *M* and its state *s*, it must decide if M⊧ϕ”. As long as *M* is finite, there will be a finite set of states and the computation will end. Unfortunately, this logic does not allow for an equality form, so it cannot be said if a transition has an inner loop or if two different actions are outgoing in the same state. In addition, it is not possible to attain either an action counter from a certain state or to a quantifier to determine if the formula is checked in each state. Fortunately, this logic has an easy aspect, a great expressive power and algorithmic properties that makes it a propositional logic for program checking.

### 2.2. Aim and Purpose

**Figure 1:**
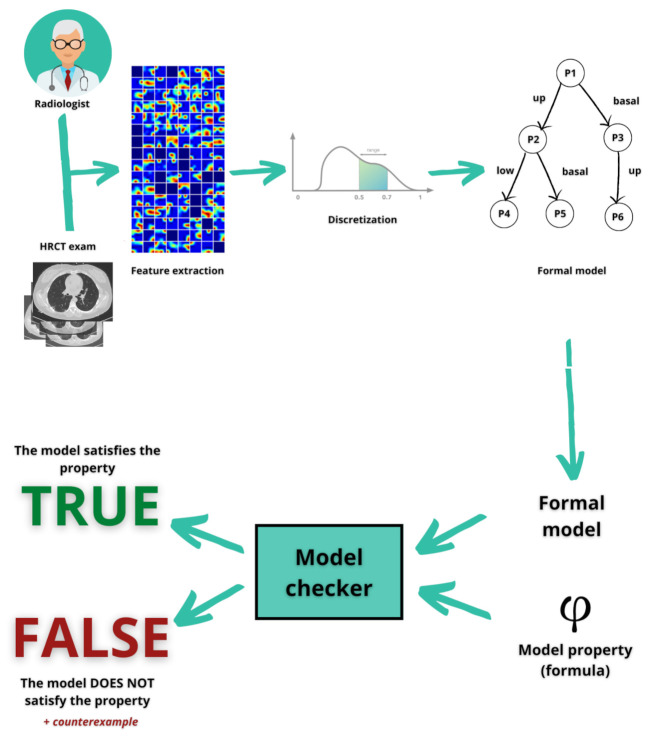
The proposed approach per automatic COVID-19 diagnosis. Starting by the medical imaging, the radiomic features are discretised and the formal model is generated, which will be evaluated by the final model-checking process.

The goal of the proposed method is to identify patients affected by the Coronavirus disease, classifying healthy, lung disease and COVID-19 patients over their medical features gained by the CT images. As shown in Figure 1, first we obtained a database of medical images of healthy and sick patients. The latter category contains different pneumonia patients and COVID-19 patients. The database comprises the Digital Imaging and COmmunications in Medicine (DICOM) images of the High resolution computerized tomography (HRCT) exam of the patient lung.

Once the dataset is obtained, the proposed method analyses the medical images by exploiting an open-source tool that helps to get numerical data from medical images. For this task it is considered the “PyRadiomics” [15] tool, able to perform the radiomic feature extraction thanks to some mathematician descriptors. There is a large number of features, hence the authors made a preliminary analysis based on the value differences of the medical status categories. Consequently, the chosen parameters will be discretised and translated in a formal language to create the formal model. This is possible through an algorithm developed by the authors that is able to extract intrinsic knowledge from the model and to produce the property of a diagnosis (sick or healthy). This is a fundamental part of the decision process because this point requires more effort in understanding a specific knowledge domain that could be afforded only by specialists. In the last part of the method, the Model Checker will check this model as a calculus and it will prove whether the patient is really sick or really healthy.

**Figure 2:**
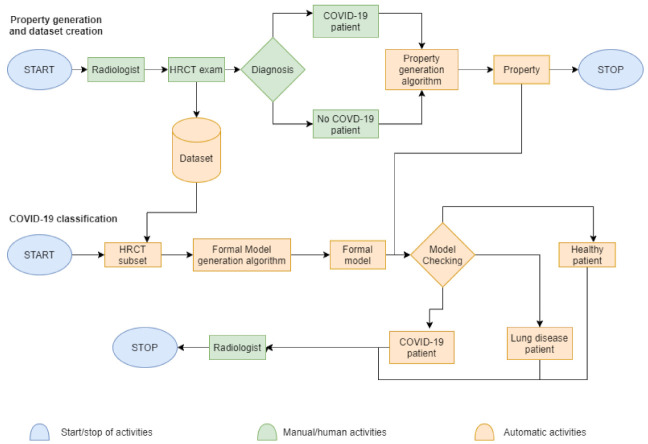
The method is above described as a block diagram divided in two processes. The first process represents the standard way a patient have to pass to do a HRCT exam and get a diagnosis. The second process, instead, exploits the first process via the complete dataset and the human knowledge of the radiologist to try to replicate the correct diagnosis.

The whole idea and method is described below in Figure 2 as a block diagram divided in two processes: on the one hand, we have the diagnosis by the radiologist and, on the other one, we have the diagnosis by machines thanks to the previous data collection.

The first process in Figure 2 is about property generation and dataset creation. The authors start thinking from the first meeting between the radiologist and patient to the final diagnosis. The second process starts from the full dataset, with which machines try to replicate the diagnosis of the specialist. The diagram legend states that the blue colour indicates the starting and the stopping of the total activities. The green represents manual or human-assisted activities, such as dealing with the radiologists, or taking the HRCT exams and getting the diagnosis by the specialist. The role of radiologist is always crucial and impossible to replace: as a matter of fact, at the end of the COVID-19 classification phase, the radiologist will certify if the computational results are equal to the previous diagnosis. The orange are all the automatic activities made by machines—the collection of HRCT images in a dataset; the creation of formal models about patients and the creation of properties representing the disease; the model checking to verify the presence of a disease.

**Figure 3:**
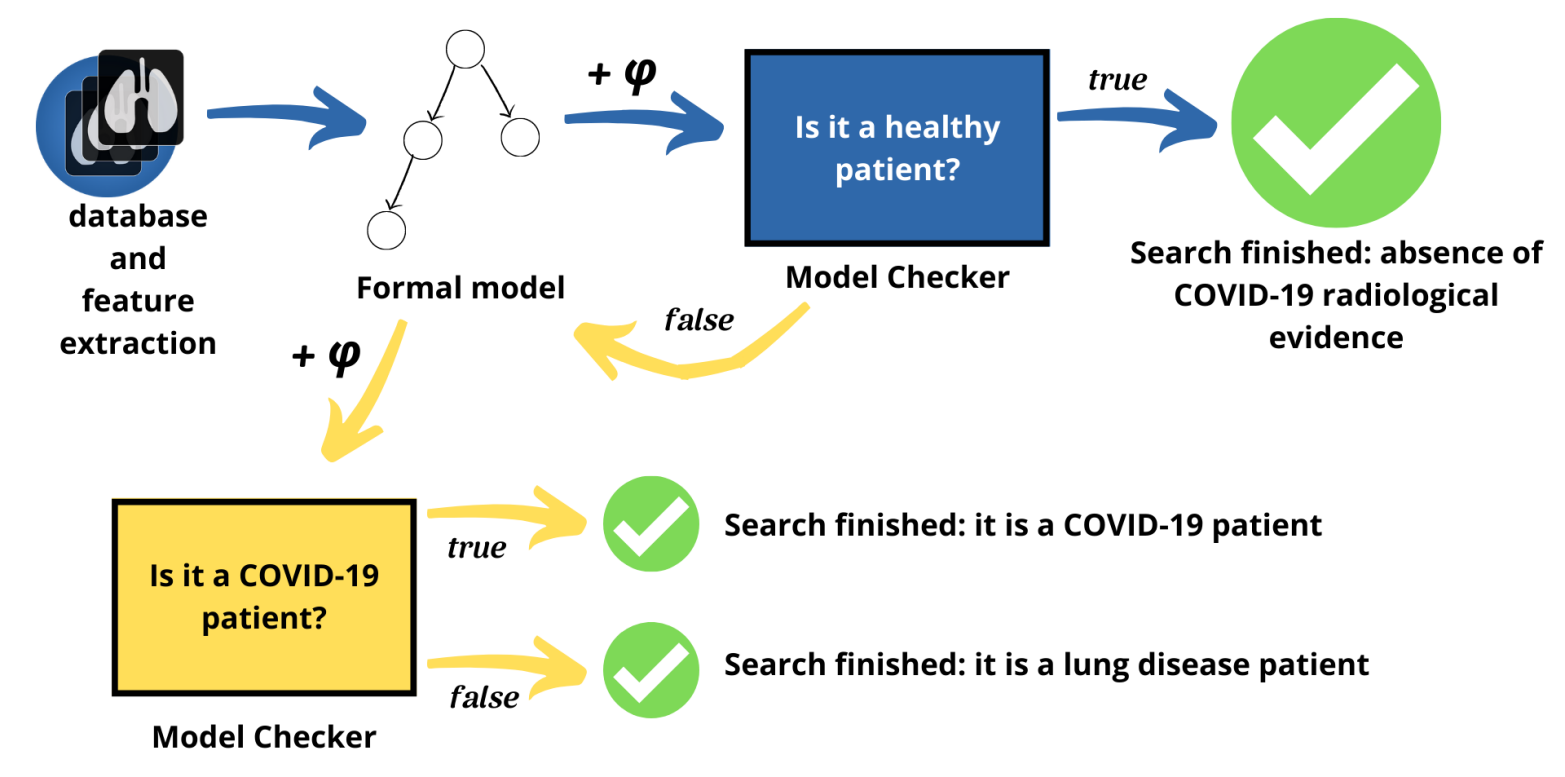
A description of the two-tier method: blue represents the classification of healthy and lung sick patients; yellow represents the classification of lung disease patients and COVID-19 patients.

To better facilitate the analysis between healthy and sick patients, the part of the method involving translation and checking is split into two steps or tiers, as shown in Figure 3. In the first step, the problem is focused on the differences between healthy and sick patients affected by any lung disease. As a result, the method can highlight the characteristics of each category. If the patient is sick, it can pass to the second step, otherwise the patient is healthy and the method can be stopped because there is no Coronavirus present. In the second step, instead, the approach deals with differences between lung disease patients and Coronavirus disease patients.

**Figure 4:**
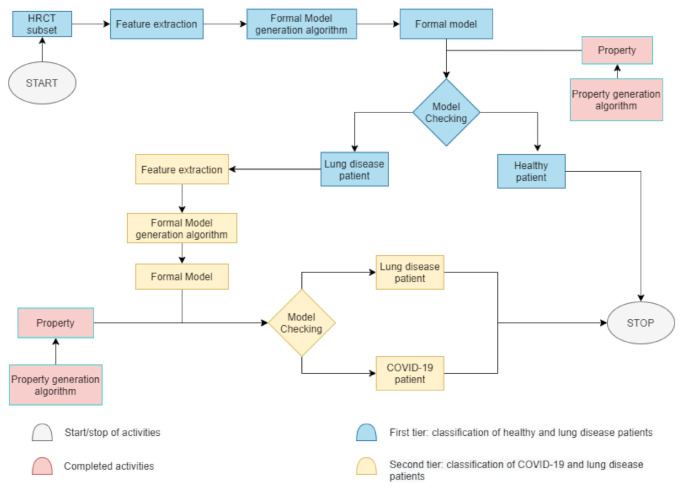
This block diagram describes the internal classification split. Grey boxes are the start and stop of activities. The pink boxes are the external activities. Property generation is already done during data collection. The blue boxes are first-tier activities, namely all activities aimed at classifying healthy or sick patients. The yellow represents the second tier, where the task is the classification between COVID-19 or non-COVID-19 patients.

To better understand the split in the proposed method, we present Figure 4, a block diagram divided in two main tiers—the first blue phase and the second yellow phase. At the beginning of the COVID-19 classification of Figure 2, in the first phase, a HRCT subset is taken. Thereafter, as shown in Figure 4, some radiomic features are extracted to automatically create a formal model, which represents the actual patient’s state of health. The property is already built during the data collection. If the property applied to the model is true, the patient is healthy and the process will stop; otherwise, the second phase begins. In the second phase, there is the need of an other feature extraction because this activity helps to concentrate the focus only on two categories of patients. If, in the first phase, the classes were healthy or sick patients, in the second phase, the task is to classify COVID-19 or non-COVID-19 patients. Later on, a new formal model will be generated and checked with a different property already built. If the property is true, the patient is affected by COVID-19, otherwise the patient is a lung disease patient. In both cases, at this point, the process will stop and the classification ends.

### 2.3. Pyradiomics and Data Analysis

**Figure 5:**
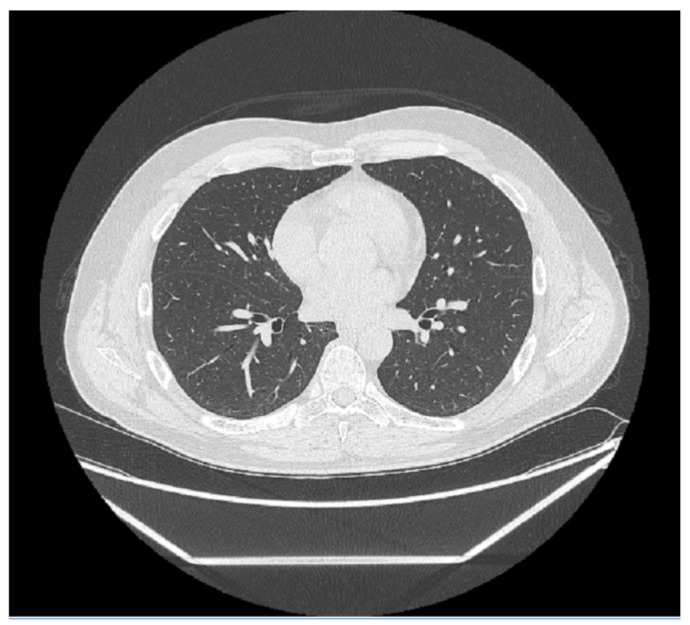
HRCT scan of a healthy patient. The image shows an example of healthy lung parenchyma.

**Figure 6:**
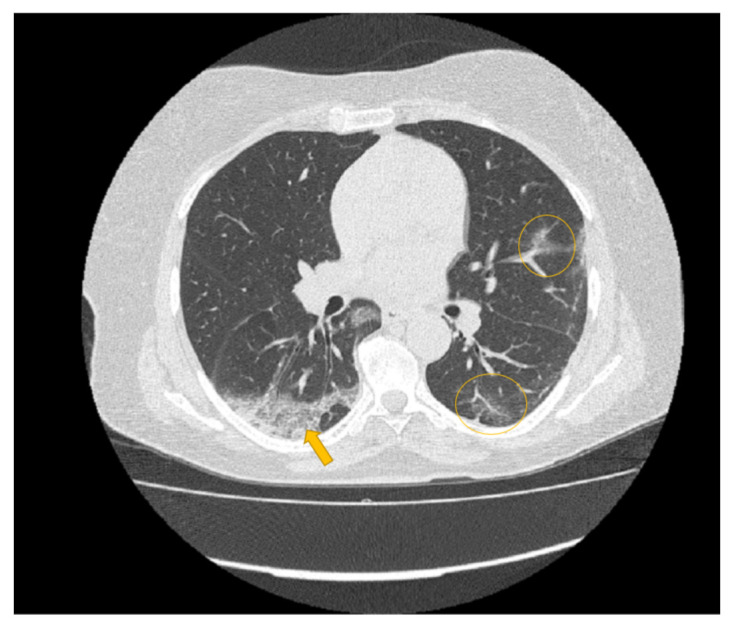
HRCT scan of a COVID-19 patient. The image revealed a lesion in the form of “crazy paving” seen in the right lung lower lobe with peripheral distribution (yellow arrow) and ground glass opacity seen in the left lower lobe (yellow circles) peripheral and central distribution.

**Figure 7:**
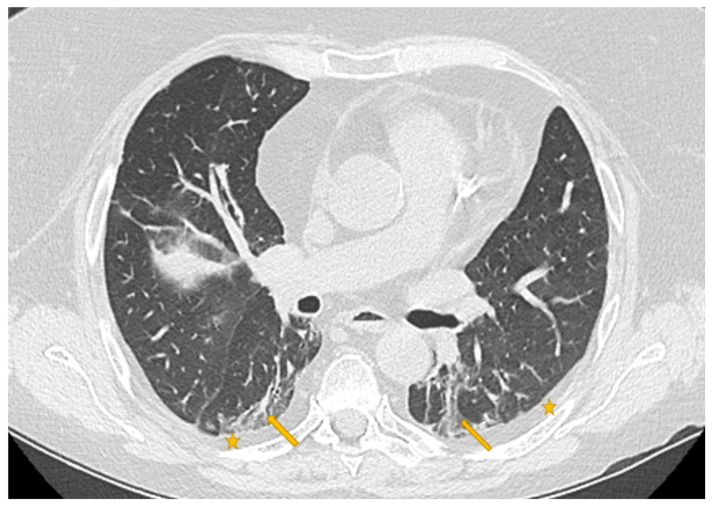
HRCT scan of a lung pneumonia. The imaging revealed lesions in the form of multiple confluent patchy areas of ground glass opacities seen scattered at both lungs (yellow arrows)—peripheral in distribution and bilateral minimal pleural effusion (yellow stars).

Every medical image is a combination of grey-levels that produce the morphology of the image. In this specific case, the attention is focused on HRCT images of the lung. In particular, the external part of a healthy lung would be white, while the inner part of the lung would be black. In this black part there could be different movements that display normal respiration and the performance of vital functions. Through these images, in the black section of the lung, radiologists can note some white spots that can be marked as disease symptoms, but they would not know to which disease the marks refer. For example, in Figure 5, Figure 6 and Figure 7 there are three different HRCT belonging to three different patients: one image has ”very black” lungs, while the others have some white spots. As a matter of fact, the Figure 5 is an HRCT of a healthy patient and it does not show white spots or pneumonia signs. The Figure 6 and Figure 7 are similar, because they both show some disease marks: the first is from a COVID-19 patient, while the second is from a lung disease patient. Thanks to this, it is clear just how difficult it is to see the differences between a general pneumonia and Coronavirus disease with the naked eye.

To perform a correct diagnosis, radiomics assists radiologists and pathologists for an objective analysis of the medical images. This is possible thanks to a Python script developed by authors for invoking the “*PyRadiomics*” [15] libraries: this open-source tool is written in Python and its goal is to extract features from medical images, starting with putting some masks on the image, called the Region of Interest (ROI), to be as effective as possible in localizing disease marks. It is generally stated that ROI localization is not an easy task. In this work, the authors did not crop the image and the ROI is considered as the whole image; namely, the whole organ: the idea is that no part of the image should be ignored in the computation. In addition, the proposed method is not time consuming and it does not require manual ROI labelling.

In *PyRadiomics* [15] there is a large number of features (estimated on statistical or mathematical calculus such as Gaussian or Logarithm, etc.) and for each slice of the HRCT exam and for each feature there is a numerical value—to improve classification results is essential in making the choice of which feature to use. As there are many images, it is difficult to see, at first glance, the important features to choose. For this reason, there is the need for a preliminary analysis of the values and the features of the different medical status categories. First of all, the authors computed all the features of each class (for a number of 107 total features) for all healthy and sick patients. Later, we compiled two lists. In the first, there was the range and the average value for healthy patients, in the second, there was the range and the average value of sick patients. These lists were compared according by the meaningfulness of a feature, namely if there were differences between the two medical status categories; i.e., if the values in the two categories are equal, the feature is discarded from both the lists. In this way, it was possible to choose less than six features for each class of features.

In the second step, this analysis was replicated, focusing the attention on the differences between COVID-19 and lung disease patients. Below, there are some features of examples taken into consideration:

- *Energy*, a measure of the magnitude of voxel values in the image. A larger values implies a greater sum of the squares of these values.
- *Gray Level Variance*, which measures the variance in grey level in the image.
- *Run Percentage*, which measures the coarseness of the texture by taking the ratio of number of runs and number of voxels in the ROI. A *run* is the length of consecutive pixels.
- *Zone Variance*, a measure of the coarseness of the texture by taking the ratio of number of zones and number of voxels in the ROI. A *zone* is the number of connected voxels that share the same gray level intensity.

These are only a few examples of the overall chosen features. In particular, the feature classes taken into account in this paper are:

- FIRST, which stands for *“First-order statistics”*. It describes the distribution of voxel intensities within the image region defined by the mask through commonly used and basic metrics.
- GLDM is the “*Gray Level Dependence Matrix*” that quantifies gray level dependencies in the image. A gray level dependency is defined as the number of connected voxels within distance δ that are dependent on the center voxel.
- GLRLM is the “*Gray Level Run Length Matrix*” and it quantifies gray level runs. *Runs* are defined as the length, in number of pixels, of consecutive pixels that have the same gray level value.
- GLSZM (used only in the first step). A “*Gray Level Size Zone*” quantifies gray level zones in an image. A *gray level zone* is defined as a the number of connected voxels that share the same gray level intensity. A voxel is considered connected if the distance is 1, according to the infinity norm.

Certainly, the preliminary analysis contributed to achieving the goal established at the beginning. The whole number of features is higher and, in the absence of numerical standard values, there are no references for healthy or sick patients. Moreover, the features are heterogeneous with each other and they are based on different mathematical filters. Nevertheless, a feature value is produced for each image slice, involving a huge dataset to analyze because each exam contains at least 200 slices. Reducing the feature dataset can allow us to concentrate on a few features; namely, on a few characteristics of the image, contributing to the *explainability* of the classification results. In this way, the authors reduced the feature dataset from 107 to less than six features. Hence, the feature sets chosen could be defined as the *best feature set*, used to fix the problem according to the tiers. For this reason, there are different feature sets in the two-tier classification. As a result, the feature sets chosen are summarized in Table 1 and Table 2.

In radiomics, there is no standard value to identify healthy or sick patients. For reasons of coherence and clarity, we show, in Table 3, an idea of the average values gained from Pyradiomics [15] in each medical status category.

### 2.4. The Method: Generation of Formal Models and Properties

Once we obtained the most important features, it was necessary to translate them into a formal language to create a formal model. The translation in LTS is obtained using the process algebra language CCS. The translation is done automatically, thanks to the algorithm *Build_LTS*. It takes the features of a slice and discretizes the values in three equal ranges (*low, basal* and *up*). Then, each feature is linked to the others of the same slice and the result is put together in a unique statement for each slice, alias as a *process*.

**Figure A1:**
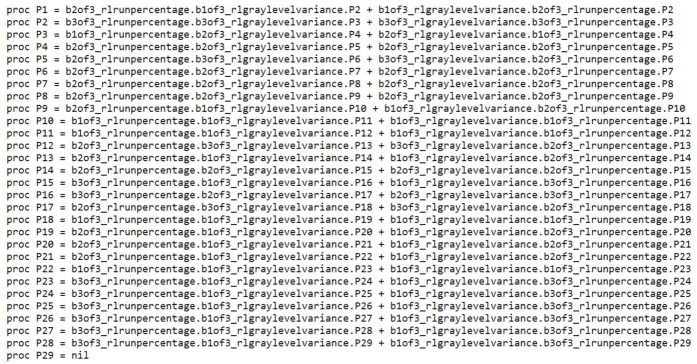
For reasons of coherence and clarity, the Figure above shows the LTS in the form of CCS processes for GLRLM Feature class. For further information, please refer to Milner’s book [11].

In the Appendix B, in Figure A1, there is an example of LTS translated in CCS logic. Each *P* is a process concatenated with others. In this case, in each process there are two radiomic features (Run Percentage and Gray Level Variance, belonging to the GLRLM class of the feature of the first step (Table 1)) that are inserted together with their range of discretization. The authors claim that a formal model represents a specific patient’s state of health in a formal language.

The same procedure happens for property generation in the *mu-calculus logic*. The algorithm *Build_Property* takes as input only two models of the same category and it generates a formula which represents the specific features of the lungs in a category. The supplied pseudocode shows some structures:

- $M_{1}$ and $M_{2}$ are two models of the same category to give as input to the algorithm, which analyze them through $index_{M1}$ and $index_{M2}$.
- candidateProperty represents the output and, therefore, the generated property.
- For each model, the sub-processes are saved in the variable $f_{i}$ which invokes the function $featureExtractor(M_{i},P_{i})$. The latter variable counts the *i-th* process of the model.

**Figure A2:**
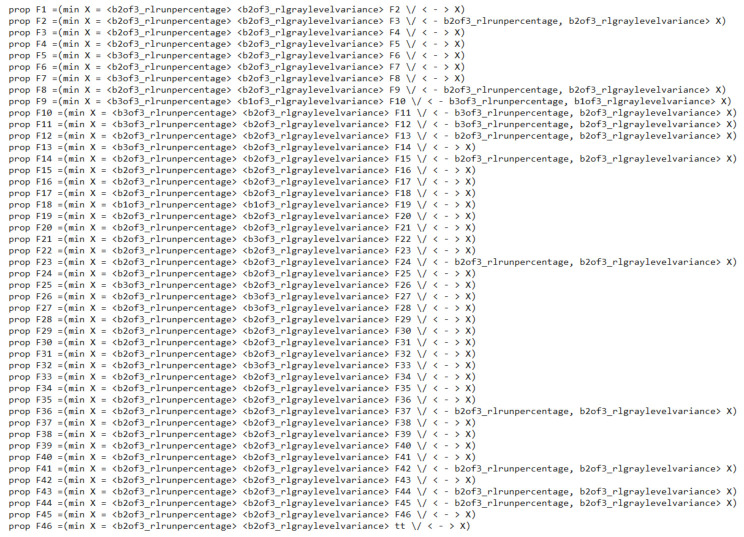
For reasons of coherence and clarity, the Figure above shows the property in the Mu-calculus algebra for GLRLM Feature class. For further information, please refer to Milner’s book [14].

Algorithm 1 starts with an empty candidate and indexes. The features are compared for each sub-process of the models. If there is a correspondence, the index is saved to lock the count and the candidate is enriched with new information. After that, the locked index will be updated and the other index will be set to exit from the comparison and from the loop. If there is never a correspondence, it means that there are no found similarities between the models and the candidate has only one standard process. In the Appendix B, in Figure A2, there is an example of medical status property automatically generated according to the Mu-calculus algebra. The appearance is similar to that of the formal model, but the property represents the main general characteristics of a medical status category (i.e., healthy patients). The radiomic features in the property are the same as those the formal model, otherwise the property will always be false because there is no correspondence. If the property finds its pattern in the formal model of a patient, the model checking decrees that the patient is affected by that medical status; namely, a diagnosis. These examples of generated LTS and property are available in the Appendix B section (Figure A1 and Figure A2).
**Algorithm 1** Build_Property pseudo code1:candidateProperty←tt2:$indexM_{1}\leftarrow 0$3:$supportM_{2}\leftarrow 0$4:**while**$indexM_{1}<getLength\left(M_{1}\right)$**do**5:    $f_{M_{1}}\leftarrow featureExtractor\left(M_{1},P_{M_{1}}\right)$6:    $indexM_{2}\leftarrow supportM_{2}$7:    **while** $indexM_{2}<getLength\left(M_{2}\right)$ **do**8:        $f_{M_{2}}\leftarrow featureExtractor\left(M_{2},P_{M_{2}}\right)$9:        **if** ($f_{M_{1}}\cap f_{M_{2}}\neq ⌀$) **then**10:           $candidateProperty\leftarrow candidateProperty+(f_{M_{1}}\cap f_{M_{2}})$11:           $supportM_{2}\leftarrow indexM_{2}+1$12:           $indexM_{2}\leftarrow getLength\left(M_{2}\right)$13:        **else**14:           $indexM_{2}\leftarrow indexM_{2}+1$15:        **end if**16:    **end while**17:**end while**

**Figure 8:**
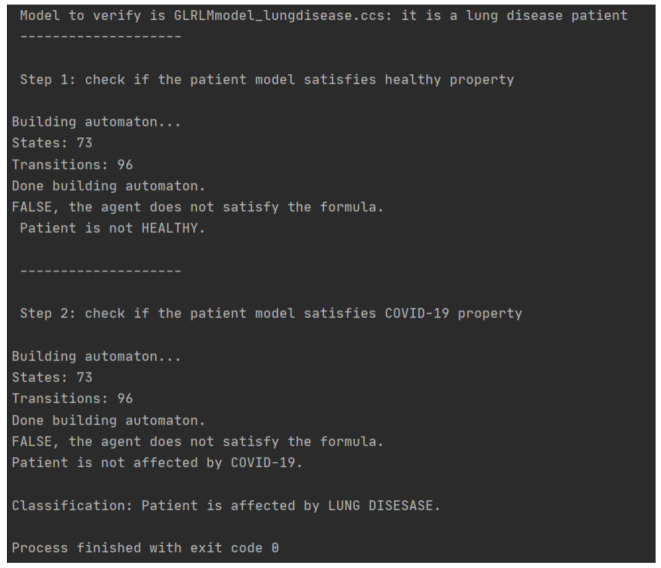
This Figure represents the execution of a diagnosis. The program starts by inputting a formal model (in this case, a lung disease model), and the property of the healthy state because we are in the first step. The result is “FALSE”, so Model checking verified that the patient is not healthy. The method will go on to the second step. Now, the program has as an input the same patient model with the COVID-19 property: the result is “FALSE”. In conclusion, the diagnosis is that the patient is affected by a lung disease.

Below, in Figure 8, there is an example of classification execution. The case study regards a patient affected by lung disease.

## 3. Results

Each HRCT exam has a number of slices of around 200–600 images. In the database used for this paper, there are 35 patients divided into three categories according to their medical status. There are nine healthy patients, six lung disease patients and 20 Coronavirus disease patients. The HRCT images are supported by the University of Molise, Campobasso, Italy and the University of Campania, Caserta, Italy.

Model checking aims to verify if the properties are satisfied by the formal models built. The model checker takes as an input two CCS models (or, equivalently, two LTSs) and a property for answering a specific question in each tier. In the first tier, the question is “Is it an healthy patient?” In the second tier, the question is “if it is not healthy, is it a Coronavirus disease patient?”. As already stated, HRCT patterns of the Coronavirus disease are nonspecific; so, for the radiologist, is so difficult to express a diagnosis of certainty. For the results, authors noted that, in the first step, the differences between patients are really discriminant (Table 4), while, in the second step, the differences between medical status categories decrease (Table 5).

Precision and recall allow us to assess the classifier performance through its *false positive* (all the true instances that are false) and its *false negative* (all the false instances that are true). In particular, Precision (Equation (A1)) (also called positive predictive value) is the fraction of relevant instances among the retrieved instances. Recall that (Equation (A2)) is the fraction of relevant instances that were retrieved. Both precision and recall are, therefore, based on relevance. Accuracy (Equation (A3)), instead, measures the overall performance. For this reason, it is necessary to quantify the mismatching of the method according to the different steps where the checking is. From the overall feature sets gathered using PyRadiomics [15], the preliminary analysis allows us to select only the features with a higher efficiency in terms of accuracy, which is calculated at the final step.

In conclusion, we stated that, on a dataset of 35 patients, radiologists manually diagnosed nine healthy patients, 20 COVID-19 patients and six lung disease patients. Each COVID-19 patient had a positive RT-PCR test; on the contrary, the other patients have a negative RT-PCR test. Starting from these diagnoses, the method was able to automatically produce formal models of the patient’s state of health and the property of a medical status category. Thanks to model checking, the results are obtained and are collected to verify the mismatching with the radiologist’s diagnosis. Obviously, the results are also compared according to the tier, because the classification works on different medical statuses and, namely, different images and values. As summarized in Table 4, in the first tier, for the classification between healthy and sick patients, we achieved an accuracy of greater than 82% of radiomic features of the First Order (values distribution analysis) and of the matrices GLRLM and GLSZM (texture analysis). In Table 5, the classification concerns COVID-19 and non-COVID-19 patients; therefore, the results do not exceed those of the first tier, but there is an improvement in terms of number of false negatives; namely, of recall or sensitivity. In this case, the most important radiomic classes are the matrices GLDM and GLRLM (about texture analysis).

Table 4 and Table 5 show the results according to the feature classes of membership.

## 4. Discussion

In the experimental analysis, the authors noted that the results reveal a situation slightly different in the first step regarding the second one. This behavior is correspondent to reality, because there is more HRCT radiological evidence between healthy and lung disease patients (Table 4) rather than in those between lung disease and COVID-19 patients (Table 5), that share many similarities. Despite this, the results in terms of accuracy are similar to the recent AI techniques, looking at all of the results and also considering the number of patients involved and the correlation with the novel techniques used. In this manner, the accuracy can be the same between two different techniques, but with different environmental conditions that involve people who use the technique or who do not. The advantage of using this method is the possibility to have an accurate, automatic, easy and quick “*second virtual opinion*” for radiologists.

In addition, we can claim that this diagnosis can also be helpful against RT-PCR tests. As a matter of fact, it is not certain that RT-PCR tests return a positive result for each patient in each disease stage. In our case study, RT-PCR tests have the maximum accuracy—i.e., each COVID-19 patient has a positive test, whereas each healthy or lung disease patient has a negative test. However, this methodology can be helpful when RT-PCR tests are negative but the patient remains a “suspect”, due to the onset of symptoms [8]. Alternatively, CT can also be performed while specialists are waiting for the RT-PCR results in cases of severe symptoms. Thus, thanks to CT exams, in both cases, model checking can be applied quickly and help in the verification of the patient’s state of health.

### 4.1. Related Work

The great importance of CT in COVID-19 recognition has been declared in the medical sector. In fact, Urciuoli and Guerriero [16] tried to replicate the CT of patients after 4 months from the onset of the pandemic. On six COVID-19 patients, they found that novel CT still contained some lung abnormalities.

Instead of using HRCT, Afat et al. [17] chose Dual-Energy Computed Tomography of the lung in 14 COVID-19 patients to study the relationship between pulmonary perfusion objects and opacities with 3D segmentation. In contrast to us, for this study, two radiologists and the Linkert scale combined with a statistical analysis were necessary. However, as declared by other researchers [2], HRCT is generally preferred for COVID-19 exams and to classify Coronavirus disease patients.

The fundamental role of the radiologist is also highlighted by Buttner et al. [18], who presented a semi-quantitative method to identify patients affected by COVID-19 in need of ICU treatment or intubation. In contrast to the method we propose, they used a database of 28 COVID-19 patients for statistical study (CoX Regression, ROC and AUC), establishing an hazard-ratio of 95%. Our approach is aimed at automatically diagnose if a patient is affected by COVID-19, while the authors [18] have to provide to radiologists and pathologists insights for disease treatment. Moreover, to better differentiate this paper with that of authors [18], their method requires image segmentation at three levels and manual ROI labeling; however, they have the ability to recognize an ICU rate of 85.6% and 71.9% for intubation.

The application of Artificial Intelligence (AI) in the medical sector for the detection of COVID-19 affected patients is present in different papers, such as that argued by Brunese et al. [19]. The paper is aimed to reach a fully automatic fast diagnosis for the new Coronavirus in X-ray images through an AI algorithm—deep learning. In a different way from us, the cited method [19] uses X-ray images, which have a lower resolution for lung disease marks.

Rajaraman et al. [20] instead introduced weakly labeled data augmentation for deep learning used in COVID-19 detection from chest X-rays. The idea is to increase training data to recognize COVID-19 opacity in the lungs using weakly labeled data augmentation. The rationale behind their method was that the COVID-19 disease is similar to other pneumonitis, such as those that are viral or bacterial. We share this assumption, but sometimes COVID-19 and pneumonia are too similar and this could lead to mistakes. Instead of high-resolution CT, they [20] used chest X-ray images to train a convolutional neural network based-algorithm, obtaining an accuracy of 0.8889%. Contrary to this paper, using AI involves the need for a huge dataset (they chosen six big data set) and for assistance in the Deep Learning model, which comprehends different onerous actions—starting to segment the images for producing the ROI, to adjust images with different steps of pre-processing, to standardize images to establish the computational effort. This is not required when using formal methods.

Pengyi Zhang et al. [21] proposed a novel conditional generative model, called CoSinGAN, to learn lung and infection segmentation with five-fold cross validation of the CT dataset. They evaluated the performing model on 20 CT cases and on the independent testing of 50 CT cases. Compared to this paper, they do not use a high resolution CT and they spent 20 cases training the model. At the same time, we use only six models to create the optimal property of one medical status.

A benefit of using HRCT and formal methods is stated by the lack of crucial X-ray images about COVID-19 to train AI models. However, Loey et al. [22] introduced a novel detection model based on GAN and Deep Transfer Learning to make up for the lack of a dataset, especially in chest X-ray images. The idea is to collect all the possible images of COVID-19 and use the GAN network to generate more images to help in the detection of this virus from the available X-ray images with the highest possibe accuracy.

As far as the authors know, there are no other studies on COVID-19 that apply formal methods or formal validation. Further similar studies to the one presented in this paper are those using similar categories of patients in the database, even if they are using different techniques and radiomics. For instance, Civit-Masot et al. [23] studied the effectiveness of the VGG-16 Deep Learning model for the identification of pneumonia and COVID-19 patients using torso radiographs. The database used is similar to our database for the medical categories included because it is a combination of healthy, Pneumonia and COVID-19 X-ray images available online (396 images for three different medical status). They did the training of the model and a pre-processing stage to standardize the images and the assessment of the testing stage using around 27 images for each category. On the contrary, we used hundreds of images for each patient, which should mean the higher the reliability of the method. Their results show an accuracy of 0.85, a precision of 0.85, a specificity of 0.92 and a sensitivity of 0.85: it may also seem that the accuracy results are similar to this paper [23] and the others.

There are many case studies that tried to study the perfect combination between AI and medical exigences. In particular, as regards the COVID-19 disease, it is necessary to mention some similar studies, such as the one conducted by Wang et al. [24] and the one by Hemdan et al. [25]. In the first work, the authors [24] do not take into account healthy patients, but they did conduct dataset collection for COVID and lung disease patients. Therefore, they randomly selected a ROI and they created a deep learning model with an accuracy of 0.82%. Other studies were performed by Song and other authors in [26]. They studied COVID disease on CT images, but they were limited in the database collection because they did not collect images of other pneumonitis, which could be very similar to Coronavirus pneumonia. As we already know, AI is already well defined in the medical field; however, our method works well on CT images and it does not need an exhaustive type of training learning model, as for deep learning models. In addition, CT images are more accurate and have a greater sensitivity, especially for the COVID-19 classification problem.

As shown in Table 6, there is no formal method approach on Coronavirus, to the best of authors’ knowledge. However, the application of formal methods in the medical field is advanced by a paper that studies prostate cancer prediction. In the assumptions stated by Brunese et al. [27], it is possible to identify the smallest cancer in MRI images and cancer forms that can be easily ignored or that can evolve in severe forms. This paper, as with our paper, contributes to the notion that formal methods have a better accuracy than those of recent AI technologies. In addition, their results [27] are comparable enough with those of this paper, despite considering a different organ and goal.

### 4.2. AI and Formal Methods

For all the AI techniques exploited in a healthcare context, an important part of the model design with medical imaging is focused on ROI localization. This is aimed at cutting the image and taking into account only that region of the image. Those “cuts” have different pros and cons—they help to concentrate the study only on smaller regions of the image and they could improve model performance. On the other hand, with the cuts, the prior model excludes some regions that will be ignored and this could be the source of many faults. For this reason, the proposed method takes into account all the images without cuts. In other words, the ROI used in this paper is the whole image.

AI is in every sector, and it was likely the first computational technique used in radiology. Novel models to help healthcare specialists are evolving day-to-day, but there is a lack of *explainability* in the decisions taken by these models. As explained by Fosca et al. [28], the last decade has shown the rise of ubiquitous opaque decision systems. AI is considered as a *black box system*, considering sophisticated machine learning models to predict individual information. This learning process produces an outcome or a decision which is learned by the traces left behind from the activities of the people; this amount of data may contain human biases and prejudices, hence the decision can be inherited and can lead to unfair and wrong decisions. For this reason, the GDPR included clauses on automate individual decision making, such as the right of *explanation* for the logic involved in the automated decision-making algorithm.

In addition, artificial intelligence techniques usually generate models in which prediction are not immediately understood from the user’s point of view. This would impact ethics, safety and industrial liability. Additionally, the scientific research, the use of machine learning requires an explanation for trust, the acceptance of results and “for the sake of the openness of scientific discovery and the progress of research” [28].

We take care with formal methods, mainly for *explainability*. Explanation is fundamental for responsible open data science across different sectors and disciplines. There is the need for *interpretable*, explainable and comprehensible models to provide meaning or to explain and present concepts in understandable terms. However, interpretability is a complicated, large and difficult concept.

As explained by Parnas [29], the “application of AI methods can lead to devices and systems that are untrustworthy and sometimes dangerous”, especially for programs aiming to imitate humans activities, such as that made by medical specialists. AI researchers describe their activities as “heuristic” methods, which means their model “does not always get the right answer” because they could be based on rules of thumb and experience, but they are not supported by theory. We may remember that computers cannot recognize something that is unintelligent from something that is; hence, verifiable and formal algorithms are preferable to heuristics, because the illusion of an intelligent computer presents a risk we should not accept, at least in the medical sector.

Table 7 shows the requirements of AI compared to formal methods. It is important to claim that the comparison is made taking into account the overall AI field and methodologies.

As declared above in Table 7, for AI there is the need for huge datasets of images and a phase of training to deeply learn the classification of characteristics. Sometimes, training can be very difficult because it can attract the method towards overfitting or underfitting. This risk is absent in formal methods. The proposed technique, indeed, has an immediate construction of the model and the item to classify is represented through its main patterns, which are quantitatively visible in the image range of values. Working with numerical values often means a preprocessing and standardization of values. This is true for both techniques. However, the formal method approach is a mathematical proof of the process that led a result. It also can not be totally automatic. In fact, formal methods are helped by human knowledge to extract more suitable classification properties, which are so specific; this can seem to be a disadvantage, but in reality this human presence contributes to facilitating the explainability of the processes that led to a result. This is possible because formal methods use Model Checking. For instance, one ”*False*” classification may produce a counterexample, which is a mathematical explanation of the reasoning behind. As it happens, the counterexample can be analyzed again to find the crucial characteristics of a classification mistake or as an explanation. The same assumption can be done on the building properties. Indeed, when there is an error of classification, the problem is commonly in the property. Hence, if it is possible to analyze the ”wrong” property, it will be possible to adjust the property and include or exclude some confusing features that led to mistakes.

## 5. Conclusions and Future Work

In conclusion, the usage of formal methods to analyze COVID-19 images is entirely original and can carry out a higher accuracy of results; this assumption is yet to be demonstrated by the application of this method on prostate cancer prediction [27]. However, it is necessary to try to adopt different diagnostic methodologies that allow us to exceed the natural limits of different algorithms of Artificial Intelligence (AI), namely:

- Need for a huge dataset to achieve higher performance.
- Wrong classification on the new cases due to small differences in the system behaviour learned by the model.
- Incapacity to explain the “reasoning” that has taken place to arrive at the results, which impacts on the algorithm’s confidence.

Through formal methods, it is possible to verify, in a mathematically rigorous way, the obtained results, and also to contribute to the *explainability* problem of AI [29] using smallest dataset. It is possible to see in the “*black box of the AI*” and to have a greater awareness of the process that has produced the results. Despite this, AI is fundamental for data analysis and, if it is used together with formal methods and other medical techniques, they can gain a greater advantage for a faster diagnosis of the COVID-19 disease. Formal Methods usage will improve analysis accuracy and it will not substitute for doctors. The diagnosis will only be proposed to the radiologists, who would then make the decision in a more safe and reliable way.

As future work, this study could be enriched with other formal models and properties to decree the severity of COVID-19 pneumonia in patients. This goal can be achieved by generating properties that describe the severity degree, instead of the characteristics of a medical status. Furthermore, as future developments, this method would also serves to know the patients prognosis, differentiating the mild cases from those for which hospitalization is necessary. This means the possibility to quickly send the patients to for most suitable therapy, saving medical resources and adding a step for the medicine of the future—*personalized medicine*.

## Figures and Tables

**Table 1:** Radiomic feature set for the first tier (or step 1).

FIRST	GLDM	GLRLM	GLSZM
Energy	Dependence non uniformity normalized	Gray level variance	Gray level variance
Interquartile range	Gray level variance	Run percentage	Large area emphasis
Skewness	Large dependence emphasis		Size zone non uniformity normalized
Total energy	Large dependence low gray level emphasis		
Variance			Zone variance

**Table 2:** Radiomic feature set for the second tier (or step 2).

FIRST	GLDM	GLRLM
90 Percentile	Dependence non uniformity	Gray level non uniformity
Kurtosis	Gray level non uniformity	Long run emphasis
Minimum	High gray level emphasis	Run length non uniformity
Skewness	Large dependence emphasis	Short run high gray level emphasis
Variance	Small dependence high gray level emphasis	

**Table 3:** Average values for healthy, COVID and lung disease patients. For healthy patients, there are only two parameters because these are the most discriminant for distinction by sick patients. In fact, these five features belong to the second-tier classification (see Table 1 and Table 2).

Features	Healthy	COVID-19	Other Disease
90 Percentile	-	−144.22	−15
Kurtosis	-	5.88	3.84
Minimum	-	−1146.39	−1023
Skewness	0.45	1.17	1.09
Variance	260,000.2	201,752.4	216,780.4

**Table 4:** Step 1: Results of the comparison between healthy and lung disease patients.

Feature	False	Precision	False	Recall	Accuracy
Class	Positive	%	Negative	%	%
FIRST	0	100	6	81.25	82.86
GLDM	1	96.15	6	80.64	80
GLRLM	1	96.15	5	83.33	82.86
GLSZM	2	92.31	4	85.71	82.86

**Table 5:** Step 2: Results of the comparison between general lung disease and COVID-19 patients.

Feature	False	Precision	False	Recall	Accuracy
Class	Positive	%	Negative	%	%
FIRST	4	80.95	3	85	78.12
GLDM	3	85	3	85	81.25
GLRLM	5	79.16	1	95	81.25

**Table 6:** State-of-the-art comparison.

Method	Images	Healthy	COVID-19	Others	Accuracy
Brunese et al. [19]	X-ray	✓	✓	✓	0.97%
Hemdan et al. [25]	X-ray	✓	✓	-	0.90%
Rajaraman et al. [20]	X-ray	✓	✓	✓	0.89%
Loey et al. [22]	X-ray	✓	✓	✓	0.85%
Civit-Masot et al. [23]	X-ray	✓	✓	✓	0.85%
Wang et al. [24]	CT	-	✓	✓	0.82%
Song et al. [26]	CT	✓	✓	-	0.86%
Our method	CT	✓	✓	✓	0.83%

**Table 7:** Pros and cons of the actual technologies helping medical specialists.

Properties	Artificial Intelligence	Formal Methods
Need for huge dataset of images	✓	
Need for preprocessing and standardisation of values	✓	✓
Need for training for learning models	✓	
Mismatching on new cases due to overfitting or underfitting	✓	
Mathematical proof and testing		✓
Facilitates the explainability of the process		✓
Need for human knowledge		✓

## Data Availability

The data presented in this study are available on request from the corresponding author. The data are not publicly available due to privacy restrictions.

## Abbreviations

The following abbreviations are used in this manuscript:

COVID-19	Coronavirus Disease 2019
CT	Computational Tomography
HRCT	High Resolution Computational Tomography
DICOM	Digital Imaging and COmmunication in Medicine
RT-PCR	transcriptase-polymerase chain reaction
AI	Artificial Intelligence
ROI	Region of interest
CCS	Calculus of communicating systems
LTS	Labeled Transition System

## Appendix A. Formula

(A1) $$Precision=\frac{truepositive}{\left(truepositive+falsepositive\right)}$$

Equation of Precision metric, used to calculate the performance of the proposed method.

(A2) $$Recall=\frac{truepositive}{\left(truepositive+falsenegative\right)}$$

Equation of Recall metric, used to calculate the performance of the proposed method.

(A3) $$Accuracy=\frac{\left(truepositive+truenegative\right)}{\left(truepositive+truenegative+falsepositive+falsenegative\right)}$$

Equation of Accuracy metric, used to calculate the performance of the proposed method.
